# Early diagnosis of sepsis-related hepatic dysfunction and its prognostic impact on survival: a prospective study with the LiMAx test

**DOI:** 10.1186/cc13089

**Published:** 2013-10-31

**Authors:** Magnus F Kaffarnik, Johan F Lock, Hannah Vetter, Navid Ahmadi, Christian Lojewski, Maciej Malinowski, Peter Neuhaus, Martin Stockmann

**Affiliations:** 1 Department of General, Visceral and Transplantation Surgery, Charité – Universitätsmedizin Berlin, Augustenburger Platz 1, 13353 Berlin, Germany; 2 Department of General, Visceral, Vascular and Paediatric Surgery, University Hospital of Wuerzburg, Oberduerrbacher Strasse 6, 97080 Wuerzburg, Germany; 3 Department of Anaesthesiology, Charité – Universitätsmedizin Berlin, Augustenburger Platz 1, 13353 Berlin, Germany

## Abstract

**Introduction:**

Liver dysfunction can derive from severe sepsis and might be associated with poor prognosis. However, diagnosis of septic liver dysfunction is challenging due to a lack of appropriate tests. Measurement of maximal liver function capacity (LiMAx test) has been successfully evaluated as a new diagnostic test in liver resection and transplantation. The aim of this study was to evaluate the LiMAx test during sepsis in comparison to biochemical tests and the indocyanin green test (ICG-PDR).

**Methods:**

We prospectively investigated 28 patients (8 female and 20 male, age range 35 to 80 years) suffering from sepsis on a surgical ICU. All patients received routine resuscitation from septic shock (surgery, fluids, catecholamines, antibiotic drugs). The first LiMAx test and ICG-PDR were carried out within the first 24 hours after onset of septic symptoms, followed by day 2, 5 and 10. Other biochemical parameters and scores determining the severity of illness were measured daily. Clinical outcome parameters were examined after 90 days or at the end of treatment. The population was divided into 2 groups (group A: non-survivors or ICU length of stay (ICU-LOS) >30 days versus group B: survivors and ICU-LOS <30 days) for analysis.

**Results:**

Epidemiological baseline characteristics of both groups were similar. Group A patients had significant lower LiMAx and ICG-PDR values than patients in group B. Determination of ICG-PDR by finger probe failed in 14.3% of tests due to insufficient peripheral pulses. Respiratory, renal and hepatic dysfunction (LiMAx and ICG-PDR) were associated with prolonged ICU-LOS. Only LiMAx <100 μg/kg/h and respiratory dysfunction were associated with increased mortality. For LiMAx <100 μg/kg/h receiver operating characteristic-analysis revealed a 100% sensitivity and 77% specificity for death.

**Conclusions:**

Sepsis-related hepatic dysfunction can be diagnosed early and effectively by the LiMAx test. The extent of LiMAx impairment is predictive for patient morbidity and mortality. The sensitivity and specificity of the LiMAx test was superior to that of ICG-PDR regarding the prediction of mortality.

## Introduction

Sepsis is a frequent infection with high mortality rates in ICUs and may occur in approximately 25% of ICU patients. Multiple organ failure caused by sepsis still remains the most frequent cause of death in intensive care [1]. Patients with septic shock develop liver failure in 19% [2] with an increase up to 50% in case of acute hypoxic hepatitis [3]. Hepatic dysfunction and liver failure indicate poor outcome in critically ill patients [4,5]. Especially early hepatic dysfunction is associated with higher in-hospital mortality [6].

The diagnosis of liver dysfunction still remains imprecise. Static tests such as serum bilirubin, alanine aminotransferase, aspartate aminotransferase and the International Normalised Ratio (INR) are widely used for clinical routine assessment of liver function and in intensive care scoring systems. But there is a lack of studies evaluating the diagnostic and prognostic value of static liver dysfunction tests on outcome in critically ill patients [7].

There is some evidence that dynamic tests are superior to static tests for assessment of liver function [8]. The plasma disappearance rate (hepatic clearance) of indocyanine green (ICG-PDR) is widely used to determine liver dysfunction in critically ill patients. Many authors showed in several studies that ICG-PDR is correlated with ICU survival [9,10]. Furthermore, ICG-PDR was superior to different static liver function tests in terms of outcome prediction and indicated liver dysfunction earlier [11,12]. However, ICG-PDR has limitations due to multiple confounding factors involved in indocyanine green (ICG) clearance [13] and to the impact of several factors such as hepatic blood flow and hyperbilirubinemia [14,15].

The new dynamic bedside test for maximal liver function capacity (LiMAx test) provides a non-invasive diagnostic tool for determining liver function in different settings. The LiMAx test determines the maximal liver function capacity based on a non-invasive breath test. The test substrate is ^13^C-labeled methacetin that is exclusively metabolized by cytochrome P450 1A2 into ^13^CO_2_ and acetaminophen. In several previous studies, the LiMAx test was successfully evaluated as a diagnostic test prior to and after liver resection and transplantation [16-19].

In this study, we aimed to evaluate prospectively the LiMAx test for diagnosis of hepatic dysfunction in a well-defined cohort of patients with sepsis and compared the results with ICG-PDR. Furthermore, we investigated the LiMAx test with regard to prediction of death in septic patients.

## Materials and methods

### Study design

Patients suffering from bacterial sepsis were included in a prospective observational study. The ethics review board of the Charité medical faculty approved the study protocol before the first patient was included. All participants or their responsible relatives gave written informed consent prior to study inclusion.

Inclusion criteria were new onset of bacterial sepsis (<24 hours) according to the current definition [20] and age within 18 to 80 years. Exclusion criteria were pre-existing advanced liver disease (for example, chronic hepatitis, liver fibrosis or cirrhosis), history of chemotherapy (within 8 weeks prior to inclusion), history of daily cortisol medication >5 mg, organ transplantation, or HIV.

All patients received routine sepsis therapy (surgery, fluids, catecholamines, antibiotic drugs) according to the current sepsis guidelines [21]. The Acute Physiology and Chronic Health Evaluation II score was determined at patient inclusion (first 24 hours) and the following parameters were obtained by clinical routine on a daily basis from day 0 to day 10: Sequential Organ Failure Assessment score, Simplified Acute Physiology Score II, serum and plasma laboratory parameters, blood gas analysis, hemodynamic parameters, body temperature, mechanical ventilation, hemodialysis and medication. Additional diagnostic procedures including the LiMAx test and ICG-PDR were performed at baseline (<24 hours after onset of sepsis) and at days 2, 5, and 10 after inclusion of the patient. The patient follow-up was limited to 90 days or end of treatment. After discharge of the patient, the duration of mechanical ventilation and hemodialysis, the length of stay (LOS) in the ICU and the different organ failures were calculated.

### Definitions of organ dysfunction

Cardiac dysfunction was defined as low cardiac output by adequate volume supply (Cardiac index determined by PiCCO™, Pulsion Medical Systems SE, Feldkirchin, Germany) requiring continuous dobutamine infusion to achieve a cardiac index ≥2.5 during days 0 to 5. Hemodynamic dysfunction was defined as arterial hypotension despite adequate volume supply requiring continuous norepinephrine infusion to achieve a mean arterial pressure ≥65 mmHg during days 0 to 5 [21]. Microcirculatory dysfunction was defined as metabolic acidosis with serum lactate >4.4 mmol/l during days 0 to 5 [22]. Respiratory dysfunction was defined as respiratory insufficiency requiring mechanical ventilation >24 hours during days 0 to 10. Renal dysfunction was defined according to the RIFLE criteria [23]. Hematologic dysfunction was defined as platelet count <100/nl [24]. Hepatic dysfunction was defined as INR >1.5, increase of serum bilirubin ≥70 μmol/l during days 0 to 10 [25-27], ICG-PDR ≤10.3%/minute during days 0 to 10 [10], and LiMAx <100 μg/kg/hour during days 0 to 10.

### LiMAx test and plasma disappearance rate of indocyanine green

The LiMAx test was performed as described previously using the FLIP™ Analyzer (Humedics GmbH, Berlin, Germany) [19]. ^13^C-labeled methacetin (Humedics GmbH) was applied by an intravenous bolus of 2 mg/kg body weight. The normal range was defined as >315 μg/kg/hour [19]. In mechanically ventilated patients a special tube was used to dissipate the exhaled gas to the ^13^CO_2_ detector.

ICG-PDR was performed by ICG pulse dye densitometry using a commercially available analyzer (DDG-2001, Dye Densitogram Analyzer; Nihon Kohden, Tokyo, Japan) and a corresponding finger probe (DDG Analyzer Finger Probe TL-301P; Nihon Kohden). ICG (ICG-Pulsion; Pulsion Medical Systems, Munich, Germany) was applied by an intravenous bolus of 0.5 mg/kg body weight. The normal range of ICG-PDR was defined as 18 to 25%/minute [9]. If patients were on continuous hemodialysis, the injection of ^13^C-methacetin and ICG was performed after interrupting the blood pump for 60 seconds to avoid recirculation and altered kinetics.

### Statistical analysis

The primary analysis was based on the combined study endpoint of negative clinical outcome, defined as sepsis-related death or ICU LOS ≥30 days (group A). In contrast, patients of group B survived sepsis and had ICU LOS <30 days. A *post-hoc* power calculation based on the initial LiMAx results (independent means in group A vs. group B; α = 0.05, two-tailed, using G*Power 3.1, Heinrich Heine University, Duesseldorf, Germany) yielded 1 – β = 0.979.

Data are presented as mean with standard deviation and range, if not otherwise noted. Individual time points were compared using repeated-measures analysis of variance. Group differences were compared by the appropriate tests according to scale and data distribution, including Fisher’s exact test, the chi-square test, and the independent *t* test. The receiver operating characteristic (ROC) curve was applied to calculate the best cutoff value of minimum LiMAx during days 0 to 10 for prediction of negative clinical outcome. Statistical significance was considered *P* <0.05. Statistical analysis was performed using IBM SPSS 19.0, Armonk, NY, USA.

## Results

Twenty-eight of a total number of 185 potential patients with new onset of sepsis were recruited during October 2011 and February 2013. The main exclusion criteria were preexisting liver disease or history of chemotherapy (*n* = 30), liver transplantation or daily cortisol medication >5 mg (*n* = 32), refusal of participation (*n* = 13) and unknown onset of sepsis or administrative reasons (*n* = 82). Sepsis was caused by secondary peritonitis (*n* = 15), pneumonia (*n* = 9) or other foci (*n* = 4). The majority of patients developed septic shock (*n* = 23, 85.7%) with multiple organ dysfunction syndrome (*n* = 22, 78.6%). The following organ systems developed dysfunction during sepsis: cardiac (*n* = 9, 32.1%), hemodynamic (*n* = 22, 8.6%), microcirculation (*n* = 9, 32.1%), respiratory (*n* = 17, 60.7%), renal injury (*n* = 14, 50%), renal failure (*n* = 11, 39.3%), hematologic (*n* = 11, 39.3%), and hepatic (*n* = 6, 21.4%). Nine patients (32.1%) required hemodialysis. Four patients (14.3%) developed acute respiratory distress syndrome. Baseline characteristics of the admission day to ICU divided into group A versus group B are presented in Table 1. Significant differences between group A versus group B were observed for the Acute Physiology and Chronic Health Evaluation II score and the Sequential Organ Failure Assessment score on admission and for epinephrine dose, pH, serum lactate, INR, LiMAx and ICG-PDR during the first day.

**Table 1:** Baseline characteristics

	**Group A (patients deceased or ICU LOS ≥30 days)**	**Group B (patients survived and ICU LOS <30 days)**	***P *** **value**^**a**^
Patients	14	14	
Age (years)	64 ± 8 (50 to 80)	67 ± 13 (35 to 80)	0.48
Gender (male/female)	11/3	9/5	0.68
Septic focus			0.36
Abdomen	7	8	
Lung	6	3	
Other	1	3	
Level of sepsis			0.34
Sepsis	0	2	
Severe sepsis	1	1	
Septic shock	13	11	
APACHE II score	34.9 ± 8.4 (18 to 49)	19.1 ± 6.4 (10 to 27)	<0.001
SAPS II score	59.1 ± 19.6 (23 to 90)	44.4 ± 16.1 (18 to 68)	0.043
SOFA score	11.4 ± 4.8 (23 to 90)	6.9 ± 3.8 (0 to 13)	0.017
Procalcitonin (ng/ml)	25.6 ± 25.6 (1.1 to 73)	20.4 ± 27.3 (0.9 to 75)	0.60
C-reactive protein (mg/l)	184 ± 72 (91 to 330)	139 ± 91 (40 to 290)	0.17
Temperature (°C)	37.2 ± 1.4 (35.0 to 39.5)	37.9 ± 0.9 (36.5 to 39.8)	0.14
WBC (/nl)	20.6 ± 16.9 (1.1 to 46.6)	10.4 ± 7.8 (1.4 to 27.3)	0.051
Heart rate (/minute)	112 ± 22 (80 to 160)	112 ± 26 (75 to 170)	0.91
MAP (mmHg)	69.8 ± 7.6 (55 to 80)	69.4 ± 7.5 (55 to 85)	0.89
Noradrenalin (μg/kg/minute)	0.42 ± 0.44 (0 to 1.50)	0.12 ± 0.09 (0 to 0.26)	0.024
Dobutamine (μg/kg/minute)	1.3 ± 2.6 (0 to 8.7)	0.3 ± 1.0 (0 to 3.8)	0.19
Lactate (mmol/l)	6.2 ± 5.5 (0.6 to 16.6)	2.4 ± 1.8 (1 to 8.2)	0.028
pH	7.25 ± 0.10 (7.05 to 7.41)	7.33 ± 0.10 (7.03 to 7.45)	0.031
Bilirubin (μmol/l)	22 ± 18 (5 to 63)	11 ± 13 (3 to 53)	0.084
INR	1.7 ± 0.32 (1.39 to 2.44)	1.35 ± 0.19 (1.13 to 1.69)	0.002
LiMAx (μg/kg/hour)	127 ± 88 (43 to 384)	289 ± 117 (101 to 469)	<0.001
ICG-PDR (%/minute)	8.8 ± 4.0 (3.4 to 16.9)	25.0 ± 8.2 (14.3 to 39.6)	<0.001

Data are presented as mean ± standard deviation (range). APACHE, Acute Physiology and Chronic Health Evaluation; ICG-PDR, indocyanine green plasma disappearance rate; INR, International Normalised Ratio; LiMAx, maximal liver function capacity; LOS, length of stay; MAP, mean arterial pressure; SAPS, Simplified Acute Physiology Score; SOFA, Sequential Organ Failure Assessment; WBC: white blood cell count. ^a^Fisher’s exact test or independent *t* test.

### Hepatic dysfunction

**Figure 1:**
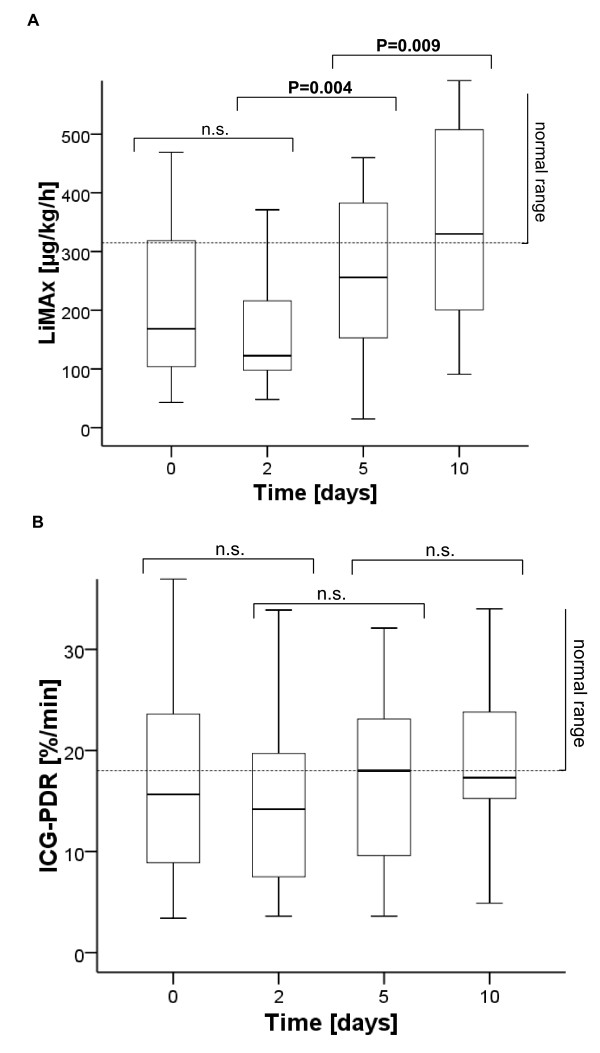
**Overall progression of dynamic liver parameters during sepsis. (A)** Maximal liver function capacity (LiMAx). **(B)** Indocyanine green plasma disappearance rate (ICG-PDR). Bold lines, medians; box plots, 25th to 75th percentiles.

The overall LiMAx values slightly dropped from baseline (208 ± 131 μg/kg/hour) until day 2 (165 ± 93 μg/kg/hour; *P* = 0.161), and then significantly recovered at day 5 (289 ± 190 μg/kg/h; *P* = 0.004) and at day 10 (357 ± 179; *P* = 0.009; Figure 1A). The ICG-PDR was constant from baseline to day 2 (17.6 ± 10.5 vs. 16.2 ± 10.3; *P* = 1.0), increased slightly at day 5 (18.1 ± 9.6; *P* = 0.44), and remained constant until day 10 (19.2 ± 8.7; *P* = 1.0; Figure 1B). The determination of ICG-PDR by pulse dye densitometry with finger probe failed during 16 examinations (14.3%) due to insufficient signal preferentially by impaired peripheral circulation (six times at baseline, three times each at day 2 and day 5, four times at day 10). All LiMAx measurements were valid. Serum bilirubin was within normal range at baseline (16.7 ± 16.3 μmol/l; median 9.4 μmol/l) and slightly increased to a maximum at day 6 (30.3 ± 42.8 μmol/l; median 10.3 μmol/l; *P* = 0.1).

**Figure 2:**
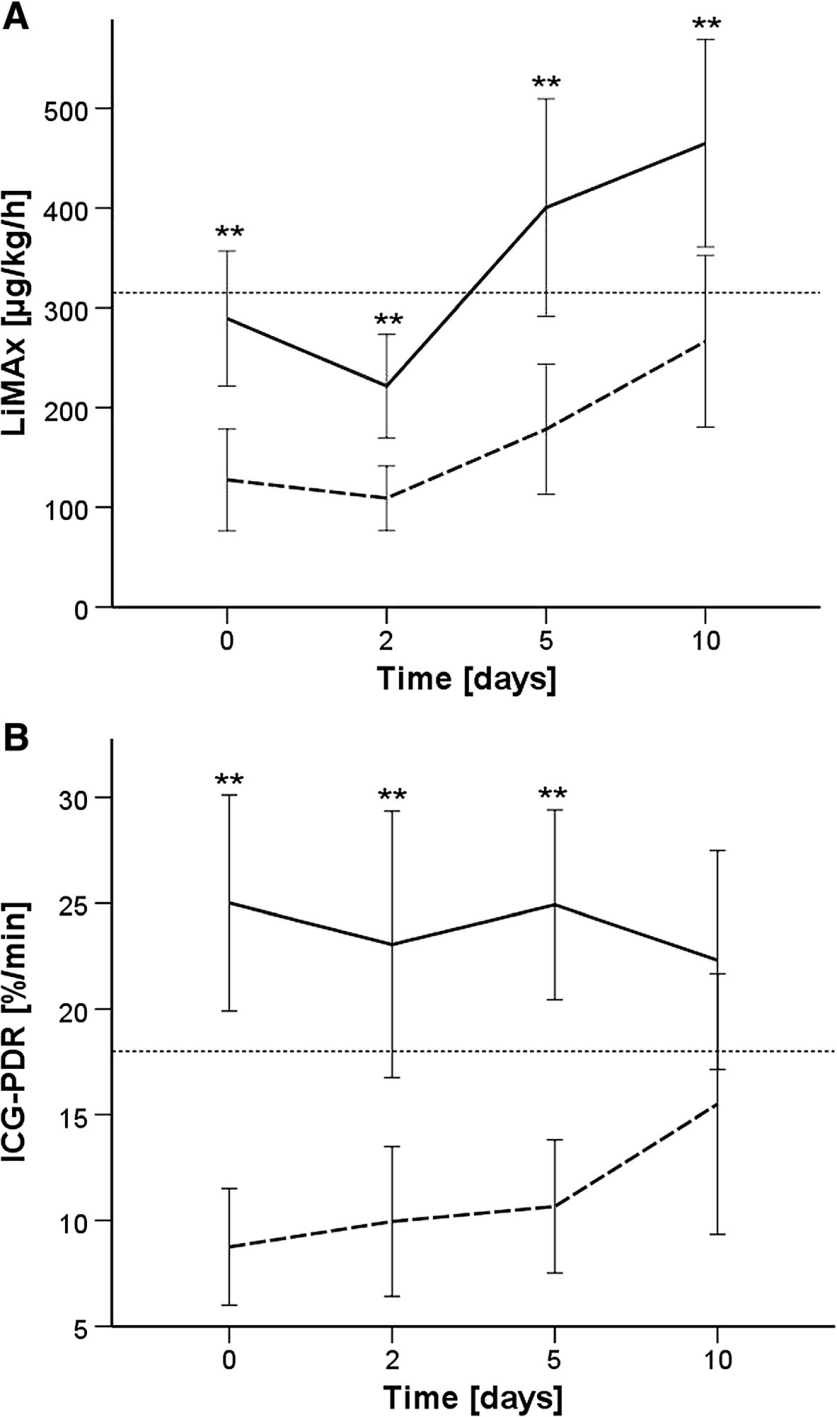
**Progression of liver parameters by patient outcome. (A)** Maximal liver function capacity (LiMAx). **(B)** Indocyanine green plasma disappearance rate (ICG-PDR). Interrupted line, group A; continuous line, group B. Mean values with 95% confidence interval. **P < 0.01.

Comparison of group A versus group B revealed significant differences for LiMAx values at baseline (*P* <0.001), day 2 (*P* <0.001), day 5 (*P* = 0.001) and day 10 (*P* = 0.004; Figure 2A), and for ICG-PDR at baseline (*P* <0.001), day 2 (*P* = 0.001), and day 5 (*P* <0.001), but not at day 10 (*P* = 0.082; Figure 2B). In contrast, serum bilirubin levels became significantly different between group A versus group B at day 2 (*P* = 0.004) and remained different until day 10 (*P* <0.05). The vast majority of baseline LiMAx values were already below normal range: overall 75%, group A 92.9% versus group B 57.1%. Baseline ICG-PDRs were below normal range in 46.4% of overall patients: group A 71.4% versus group B 21.4%. In contrast, pathological levels of serum bilirubin were observed in 25% of patients at baseline (group A 42.9% vs. group B 7.1%) and the cumulative incidence during 10 days was 50% (group A 78.6% vs. group B 21.4%). The incidence of hepatic dysfunction was therefore variable upon its definition (21.4% by serum bilirubin, 35.7% by ICG-PDR, 39.3% by LiMAx, 53.6% by INR).

### Length of stay and mortality

The ICU LOS was 33 ± 22 days (range 11 to 93 days). Six patients (21.4%) died during 7 to 52 days after onset of sepsis. In contrast, patients surviving sepsis were discharged within 11 to 93 days. The impact of the particular organ dysfunctions on ICU LOS and mortality is presented in Table 2. Occurrence of respiratory, renal or hepatic dysfunction (defined by LiMAx and ICG-PDR) was associated with prolonged ICU LOS. Occurrence of respiratory or hepatic dysfunction (only LiMAx) was associated with increased mortality. The mortality was 0% in those patients without hemodynamic, respiratory or hepatic dysfunction (defined by LiMAx).

**Table 2:** Impact of organ dysfunction on ICU LOS and mortality

**Dysfunction**	**Definition**	**n (%)**	**LOS (days)**^**a**^	***P *** **value**^**b**^	**Mortality (%)**^**a**^	***P *** **value**^**b**^
Cardiac	Low cardiac output requiring dobutamine	9 (32.1)	34 ± 24 vs. 33 ± 21	0.87	44.4 vs. 10.5	0.064
Hemodynamic	Hypotension requiring norepinephrine	22 (78.6)	37 ± 23 vs. 19 ± 10	0.067	27.3 vs. 0.0	0.20
Microcirculatory	Serum lactate >4.4 mmol/l	9 (32.1)	34 ± 24 vs. 32 ± 22	0.85	44.4 vs. 10.5	0.064
Hematologic	Platelet count <100/nl	11 (39.3)	40 ± 24 vs. 28 ± 20	0.18	27.3 vs. 17.6	0.44
Respiratory	Respiratory insufficiency requiring ventilation >24 hours	17 (60.7)	41 ± 24 vs. 21 ± 10	0.009	35.3 vs. 0.0	0.033
Renal injury	Serum creatinine >212 μmol/l or twofold increase	14 (50)	40 ± 26 vs. 27 ± 15	0.121	28.6 vs. 14.3	0.32
Failure	Serum creatinine >318 μmol/l or threefold increase	11 (39)	46 ± 25 vs. 25 ± 14	0.021	36.4 vs. 11.8	0.14
Hepatic	Bilirubin ≥70 μmol/l	6 (21.4)	47 ± 31 vs. 29 ± 17	0.24	33.3 vs. 18.2	0.38
	INR >1.5	15 (53.6)	40 ± 25 vs. 26 ± 14	0.09	26.7 vs. 15.4	0.40
	ICG-PDR ≤10.3%/minute	10 (35.7)	48 ± 21 vs. 25 ± 17	0.005	40.0 vs. 11.1	0.098
	LiMAx <100 μg/kg/hour	11 (39.3)	44 ± 19 vs. 26 ± 21	0.028	54.5 vs. 0.0	0.001

ICG-PDR, indocyanine green plasma disappearance rate; INR, International Normalised Ratio; LiMAx, maximal liver function capacity; LOS, length of stay after onset of sepsis. ^a^Presence versus absence of organ failure. ^b^Independent *t* test. ^b^Fisher’s exact test.

**Figure 3:**
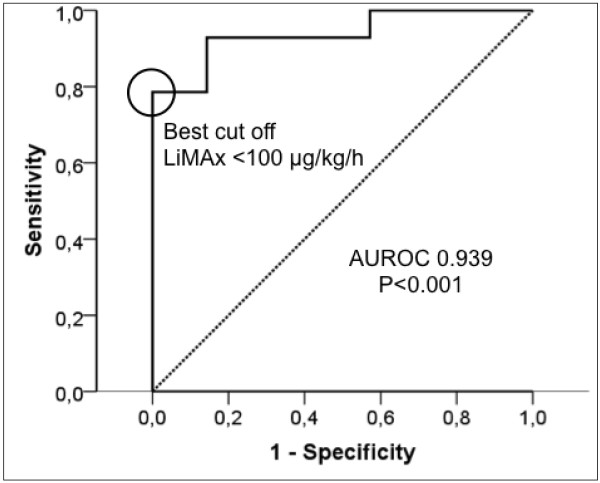
**Receiver operating characteristic curve for prediction of negative outcome by maximal liver function capacity test.** Sensitivity and specificity of maximal liver function capacity (LiMAx) <100 μg/kg/hour for prediction of negative outcome (patients deceased or ICU length of stay ≥30 days). AUROC, area under the receiver operating characteristic curve.

**Figure 4:**
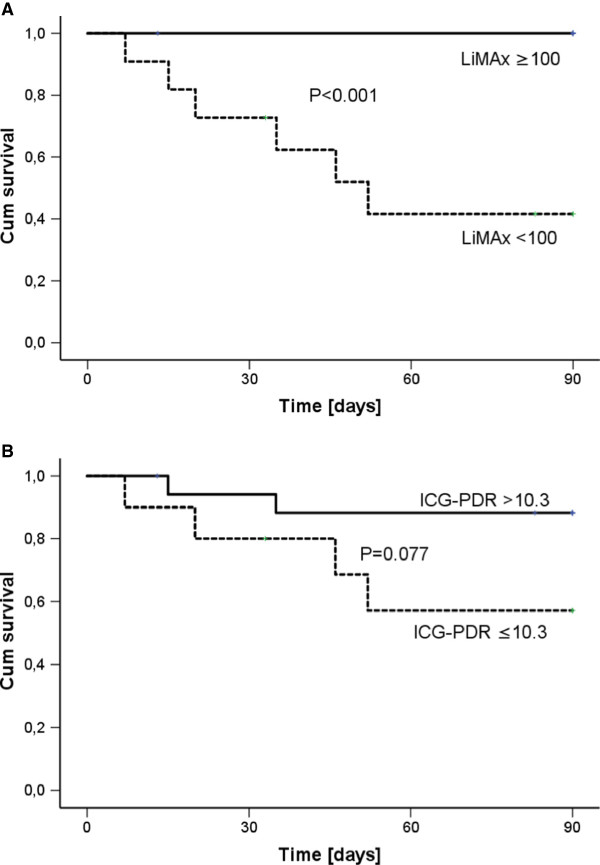
**Survival and hepatic dysfunction.** Kaplan–Meier curve for 90-day patient survival divided by **(A)** minimum maximal liver function capacity (LiMAx) <100 μg/kg/hour and **(B)** minimum indocyanine green plasma disappearance rate (ICG-PDR) ≤10.3%/minute. Log-rank test was applied for analysis.

The ROC analysis yielded a best cutoff value of 100 μg/kg/hour for prediction of negative outcome (area under the ROC curve = 0.939; *P* <0.001). The sensitivity for negative outcome was 78% with a specificity of 100%. Three patients without hepatic dysfunction but all patients without hepatic dysfunction during the initial 10 days had a prolonged LOS. The positive predictive value for negative outcome (group A) was 100% with a negative predictive value (group B) of 82% (Figure 3). All patients who died from sepsis (*n* = 6) but none of the survivors revealed LiMAx values <100 μg/kg/hour during 10 days after onset of sepsis (Figure 4A). LiMAx values <100 μg/kg/hour thus also predicted death with 100% sensitivity and 77% specificity. The positive predictive value was 54%; the negative predictive value was 100%. Two patients developed LiMAx values <100 μg/kg/hour at baseline, another two patients at day 2 and each one patients at day 5 and at day 10, respectively. In contrast, ICG-PDR was associated with prolonged LOS but did not predict survival (Figure 4B).

### Recovery of liver function

**Figure 5:**
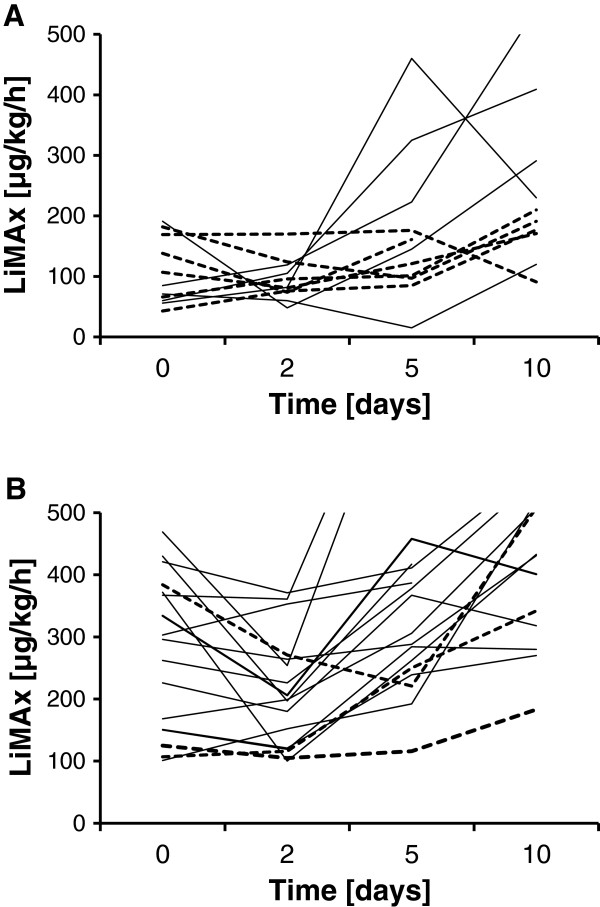
**Maximal liver function capacity progression of individual patients. (A)** Patients with minimum maximal liver function capacity (LiMAx) < 100 μg/kg/hour. Bold lines, patients who survived; interrupted lines, patients who died from sepsis. **(B)** Patients with minimum LiMAx ≥100 μg/kg/hour. Bold lines, patients of group B (survived and ICU length of stay (LOS) <30 days); interrupted lines, patients of group A (deceased or ICU LOS ≥30 days).

Those patients (*n* = 5) who survived sepsis despite single LiMAx values <100 μg/kg/hour revealed a significant functional recovery in comparison with those patients who deceased on day 5 (234 ± 170 vs. 123 ± 37; *P* = 0.032) and on day 10 (318 ± 163 vs. 168 ± 45; *P* = 0.042; Figure 5A). However, two surviving patients showed exceptional LiMAx progressions: One patient yielded functional recovery on day 5 but decreased again on day 10. This patient suffered from esophageal perforation and developed postoperative anastomosic leakage accompanied with a second septic episode starting on day 8 with increase of procalcitonin. A second patient yielded LiMAx values <100 μg/kg/hour during days 0 to 5, but thereafter the LiMAx increased slowly. This patient suffered from a severe sepsis with four-organ failure. The serum bilirubin increased up to 106 μmol/l on day 8. The patient ultimately recovered and was discharged after 77 days. In contrast, the majority of patients with LiMAx values >100 μg/kg/hour yielded fast recovery of liver function already on day 5. Those three patients with prolonged ICU LOS despite absence of hepatic dysfunction yielded either borderline dysfunction with decelerated recovery or prolonged deterioration of hepatic function until day 5 (Figure 5B).

## Discussion

The present study investigated the LiMAx test for diagnosis of sepsis-related hepatic dysfunction for the first time. The results clearly show that septic shock leads to pathologic deterioration of LiMAx within 2 days after onset of sepsis in the vast majority of patients. Furthermore, the LiMAx test indicates functional recovery within 10 days, while ICG-PDR and serum bilirubin levels remain almost constant during that period of time. There is also evidence that the degree of functional impairment determined by LiMAx is closely related to the patients’ prognosis. Patients with negative clinical outcome revealed significantly decreased LiMAx values in comparison with those patients who quickly recovered. Patients with LiMAx values <100 μg/kg/hour revealed a mortality rate of 55%, whereas patients with values >100 μg/kg/hour had a mortality of 0%. In addition, follow-up LiMAx allowed the determination of individual progress of the patients.

Since the LiMAx test had already provided promising results for the assessment of liver function in liver resection and transplantation [16-19], the aim of this study was to explore its diagnostic potential during sepsis. The well-described ICG-PDR was applied as a comparator. Although both tests are known as dynamic liver function tests, the LiMAx test determines the metabolic capacity based on the cytochrome P450 system (1A2), whereas ICG-PDR reflects the elimination of ICG into the bile, which is a transport function and not a metabolic function.

Patients were asked to participate in the study directly after onset of septic symptoms. However, the time interval between onset of sepsis and the testing by LiMAx and ICG-PDR was limited to 24 hours for administrative issues. The dynamic liver function tests were repeated after 2, 5 and 10 days to elude the progression of liver function in that crucial period. The aim was to compare liver function parameters with the clinical outcome, in particular mortality and the ICU LOS.

The LiMAx values yielded an interesting progression during 10 days. The wide range of test readouts at the baseline visit might be attributable to the variable interval from onset of symptoms to testing. The impact of sepsis, systemic inflammation and hypotension seems to have a very early effect on metabolic liver function. This effect cannot be described by biochemical parameters such as serum bilirubin, which is well within normal range at that time point and reaches maximum values as late as after 6 days. Furthermore, the early impairment of ICG-PDR might be mainly caused by impaired hepatic blood flow due to hypotension. Interestingly, the majority of patients yielded normal ICG-PDR results during sepsis, whereas 90% of patients had impaired LiMAx readouts. Stehr and colleagues described similar results in a model of septic porcines, where a discrepancy between ICG-PDR (normal) and biliary ICG excretion (decreased) was observed. These authors assumed complex ICG kinetics, especially in patients with liver disease, and temporary redistribution of ICG into extrahepatic–extravascular tissues as reasons for a false high ICG-PDR in sepsis. They came to the conclusion that ICG-PDR may not accurately measure liver dysfunction in sepsis [15]. The further progression of LiMAx yielded a nadir within 2 days followed by a fast recovery within 10 days, when the majority of patients had regained normal function. This finding confirms previous studies describing the fast functional recovery after liver resection [19]. The LiMAx test thus appears to be superior to follow-up liver function in comparison with ICG-PDR and static parameters such as serum bilirubin.

The comparison of liver function parameters and clinical outcome showed that both LiMAx and ICG-PDR impairment predicted prolonged ICU LOS, but only LiMAx values <100 μg/kg/hour predicted mortality. Serum bilirubin and INR failed to differentiate between deceased and surviving patients in terms of ICU LOS and survival. These findings confirm the validity of the LiMAx test due to prediction of mortality in patients with liver failure. Interestingly, the chosen cutoff value in sepsis seems comparable with that described after liver resection, where values <100 μg/kg/hour were associated with a very high mortality rate [17].

In our study population, serum bilirubin levels failed to differentiate between survivors or nonsurvivors/ICU LOS >30 days at any time point of the 10 days before discharge or death. These results are in line with similar contributions related to the impact of bilirubin levels in septic patients [9,26]. In a prospective study, Kramer and colleagues found early hyperbilirubinemia (bilirubin >34.2 μmol/l in the first 48 hours) to be a strong independent risk factor for mortality. In the large study population, only 11% of patients developed early hyperbilirubinemia [6]. Our data support the evidence that early liver dysfunction without evidence in clinical routine parameters occurs more frequently in the course of sepsis. These findings are in concert with the results of Recknagel and colleagues. The authors described an impaired hepatic biotransformation within hours after onset of sepsis and cholestasis (hyperbilirubinemia) as an infrequent and late event in sepsis [28]. The initial low LiMAx results in our study confirm these findings. Sakka and colleagues found in ROC analysis a higher area under the curve with respect to outcome for the ICG-PDR when using the lowest value in critically ill patients [10]. Other authors confirmed these results in septic patients and in patients after liver transplantation [29,30]. ROC statistics of our study according to mortality show a higher sensitivity of the LiMAx test (100%) compared with the ICG-PDR (70%) with a similar specificity (77% vs. 80%) [10]. The LiMAx test thus seems to be equal to the ICG-PDR test in detecting early liver dysfunction in sepsis; and among all investigated liver function tests in the present study, LiMAx <100 μg/kg/hour appears the most reliable parameter to predict mortality.

The reduction of hepatic blood flow is still considered the main cause of liver failure in sepsis, and liver failure is traditionally considered a late manifestation of sepsis-induced multiple organ failure [7]. In the past years, several studies described septic transformation in hepatic function besides an impaired hepatic blood flow. Carcillo and colleagues demonstrated a reduced detoxification system of several cytochrome P enzymes in septic patients [31]. Recent data implicate the importance of early detection of liver failure in sepsis. Recknagel and colleagues described in a rodent model the impact of impaired hepatobiliary transport and a defect in the hepatic detoxification system, including the cytochrome P450 enzyme family, with the result of a reduced xenobiotic biotransformation. This transformation will lead to impaired detoxification of substances that are normally bile excreted [28]. These findings give evidence that not only a decreased hepatic blood flow causes liver failure in sepsis. Also, processes involving the hepatobiliary transport and the biotransformation based on the cytochrome P450 family may predict the progression of liver failure in septic patients. To evaluate the impact of these changes, the metabolism of methacetin by cytochrome P450 1A2 may be helpful to quantify the degree of liver failure. From a methodological point of view it is unlikely that changes in liver perfusion above the level where cellular functional alterations takes place influence the LiMAx result since no clearance is measured apart from the formation of a metabolic product. However, actually this question cannot be answered systematically based on solid experimental data. On the other side, it is well known that the ICG-PDR not only describes the liver function but is severely influenced by the splanchnic perfusion [32]. ICG-PDR uptake and elimination in the liver is based on a carrier transport and do not reflect the changes in cytochrome P450 [33,34]. Moreover, ICG elimination is inhibited by hyperbilirubinemia (>56 μmol/l) and other anionic substances and leads to false lower ICG-PDR results [34,35] Furthermore, ICG-PDR is influenced by acute cholestasis without evidence of changes in hemodynamic or morphology of hepatozytes and, as mentioned above, by complex ICG kinetics and a temporary redistribution of ICG into extrahepatic, extravascular tissues [15,36,37]. The LiMAx test may therefore provide a more sophisticated tool to detect early liver dysfunction in critically ill patients.

Transformation of these findings into clinical routine or decision-making is a challenge. The patient population in our study is too small to establish clinical guidelines. Furthermore, no specific therapies improving the hepatic function in patients with septic liver failure are currently established. The main effort of clinicians should focus on identifying and reversing the underlying disease and stabilize the hepatic function as early as possible [38,39]. To improve the hepatic function, hepatotoxic drugs and therapies should be avoided and in special cases an extracorporeal liver support may be helpful. Thus, when LiMAx <100 μg/kg/hour during sepsis resuscitation occurs, it may indicate a poor prognosis and warns the clinician of the need for more extreme interventions.

However, the impact of hepatic dysfunction on therapy during sepsis may be underestimated at present. Especially the pharmacokinetics of many predominantly hepatic metabolized drugs remains unclear (for example, antibiotics, antimycotics). Kruger and colleagues described increased drug-levels of atorvastatin in septic patients [40]. Based on the increasing concern about toxicity of administered drugs in patients with organ failure, early detection of liver failure may become an influence on drug dosage, when plasma levels of drugs would be measured [41].

The conclusions of the present study are somehow limited due to the relative small patient population and the fact that most patients suffered from peritonitis. In addition, the LiMAx cutoff value was *post-hoc* chosen and requires prospective confirmation. Since all tests were performed after the onset of sepsis, the individual values before sepsis remain unknown. In addition, no data are available about potential long-term effects on liver function beyond 10 days after onset of sepsis. Furthermore, the determination of ICG-PDR failed in 14% of examinations due to insufficient peripheral pulses using the finger probe. Potentially the application of a nasal septum sensor might have reduced the number of missing values. Previous ICG-PDR studies investigating critically ill patients did not report any number of test failures [9,10,42].

## Conclusions

The results of this study indicate that the LiMAx test provides an adequate tool to determine early liver dysfunction in sepsis and was found to be a good predictor of survival in septic patients. The LiMAx test is equal or superior (due to prediction of death) to the ICG-PDR and superior to established static liver function tests. Because of the cytochrome P450 metabolism of the test substrate methacetin, the LiMAx test may detect liver dysfunction in sepsis earlier and better reflect the sophisticated processes triggered by sepsis in the liver.

## Key messages

•The LiMAx test, a new dynamic liver function test, is an adequate tool for early detection of liver function in septic patients.

•The LiMAx test is equal to the ICG-PDR in terms of detection of liver function and is superior due to prediction of death in septic patients.

•LiMAx <100 μg/kg/hour during sepsis resuscitation may indicate a poor prognosis.

•Cytochrome P450 metabolism of the test substrate methacetin may reflect better the complex, sepsis-triggered processes in the liver in contrast to ICG elimination, based on carrier transport.

## Abbreviations

ICG: Indocyanine green; ICG-PDR: Indocyanine green plasma disappearance rate; INR: International Normalised Ratio; LiMAx: Maximal liver function capacity; LOS: Length of stay; ROC: Receiver operating characteristic.

## Competing interests

MS is the inventor of the LiMAx test and has capital interest in Humedics GmbH (Berlin, Germany), the company marketing the LiMAx test. The remaining authors declare that they have no competing interests.

## Authors’ contributions

MFK and JFL conceived of the study and are responsible for the study design, data collection and statistical analysis. JFL performed the statistical analysis and substantially revised the manuscript. HV and NA included the patients, collected data, performed the database and statistical analysis and revised the manuscript. CL and MM recruited patients, collected data, interpreted the data and revised the manuscript. MM supervised the performance of the LiMAx test. PN participated in the study design, revised the manuscript and substantially intepreted the data of the work. MS participated in the study design, supervised the study and considerably revised the manuscript. MFK drafted the manuscript and all authors revised it critically for important intellectual content. All authors agree to be accountable for all aspects of the work in ensuring that questions related to the accuracy or integrity of any part of the work are appropriately investigated and resolved. All authors read and approved the final manuscript.
